# RGD-Targeted Ultrasound Contrast Agent for Longitudinal Assessment of Hep-2 Tumor Angiogenesis *In Vivo*

**DOI:** 10.1371/journal.pone.0149075

**Published:** 2016-02-10

**Authors:** Qiao Hu, Xiao-Yan Wang, Li-Ke Kang, Hai-Ming Wei, Chun-Mei Xu, Tao Wang, Zong-Hua Wen

**Affiliations:** 1 Department of Diagnostic Ultrasound, the People’s Hospital of Guangxi Zhuang Autonomous Region, Nanning, China; 2 Department of Pathology, the People’s Hospital of Guangxi Zhuang Autonomous Region, Nanning, China; 3 Department of Otolaryngology-Head & Neck Surgery, the People’s Hospital of Guangxi Zhuang Autonomous Region, Nanning, China; IDI, Istituto Dermopatico dell'Immacolata, ITALY

## Abstract

**Objective:**

To prepare arginine-glycine-aspartate (RGD)-targeted ultrasound contrast microbubbles (MBs) and explore the feasibility of their use in assessing dynamic changes in αvβ3 integrin expression in a murine model of tumor angiogenesis.

**Methods:**

RGD peptides were conjugated to the surfaces of microbubbles via biotin-avidin linkage. Microbubbles bearing RADfK peptides were prepared as controls. The RGD-MBs were characterized using an Accusizer 780 and optical microscopy. The binding specificity of the RGD-MBs for ανβ3-expressing endothelial cells (bEnd.3) was demonstrated *in vitro* by a competitive inhibition experiment. In an *in vivo* study, mice bearing tumors of three different stages were intravenously injected with RGD-MBs and subjected to targeted, contrast-enhanced, high-frequency ultrasound. Subsequently, tumors were harvested and sectioned for immunofluorescence analysis of ανβ3 expression.

**Results:**

The mean size of the RGD-MBs was 2.36 ± 1.7 μm. The RGD-MBs showed significantly higher adhesion levels to bEnd.3 cells compared to control MBs (*P* < 0.01). There was rarely binding of RGD-MBs to αvβ3-negative MCF-7 cells. Adhesion of the RGD-MBs to the bEnd.3 cells was significantly inhibited following treatment with anti-alpha(v) antibodies. The quantitative acoustic video intensity for high-frequency, contrast-enhanced ultrasound imaging of subcutaneous human laryngeal carcinoma (Hep-2) tumor xenografts was significantly higher in small tumors (19.89 ± 2.49) than in medium tumors (11.25 ± 2.23) and large tumors (3.38 ± 0.67) (*P* < 0.01).

**Conclusions:**

RGD-MBs enable noninvasive *in vivo* visualization of changes in tumor angiogenesis during tumor growth in subcutaneous cancer xenografts.

## Introduction

Angiogenesis, the process of blood vessel formation and recruitment, is directly associated with malignancy and necessary for cancer survival and progression [1,2]. Various molecular markers, such as ανβ3 integrin, are selectively overexpressed on the surfaces of tumor vascular endothelial cells and play crucial roles in the angiogenic process by mediating the adhesion of circulating cells to blood vessel walls and the migration of endothelial cells [3,4]. Molecular imaging of endothelial markers of tumor angiogenesis is important not only in assessing the prognosis and metastatic potential of a tumor but also in evaluating tumor response to adjuvant antiangiogenic therapies [5]. Contrast-enhanced, high-frequency ultrasonography is an attractive imaging method for the characterization of tumor angiogenesis, arteriosclerosis, thrombosis, lymph nodes, and inflammation [4,6–10]. It has advantages of wide availability, non-invasiveness, high spatial resolution, lack of ionizing radiation, and real-time anatomic visualization. When targeted ultrasound contrast microbubbles are administered intravenously, they distribute throughout vascular space and adhere to tissue sites expressing specific molecular markers, thereby enhancing imaging signals. One molecular targeting strategy is the conjugation of specific ligands, such as peptides, monoclonal antibodies, glycoproteins, and other small molecules, to microbubble shell surfaces [1].

Over the past decade, several arginine-glycine-aspartate (RGD)-based ligands have been tested for the detection of ανβ3 integrin. These ligands have been successfully visualized by molecular imaging in oncological and cardiovascular disease models [6,11–13]. Furthermore, the functionality and feasibility of covalently coupling RGD peptides to microbubbles for ultrasound-guided molecular imaging of αvβ3 integrin have been demonstrated [6]. However, few studies have reported the use of targeted, contrast-enhanced ultrasound imaging to evaluate dynamic changes in αvβ3 integrin expression at different tumor stages. Thus, in the present study, we prepared an RGD-targeted microbubble ultrasound contrast agent and explored its capacity to detect dynamic changes in αvβ3 integrin expression in a Hep-2 mouse tumor model.

## Materials and Methods

### *In Vitro* Studies

#### Preparation of Targeted RGD-MBs

RGD-MBs were prepared as previously described by Yan et al. [14]. First, 1,2-distearoyl-sn-glycero-3-phosphatidylcholine (DSPC), 1,2 distearoyl-sn-glycero-3-phosphoethanolamine-N-[methoxy(polyethylene glycol)-2000] (DSPE-PEG2000) and 1,2-distearoyl-sn-glycero-3-phosphoethanolamineN-[biotinyl(polyethylene glycol)2000] (DSPE-PEG2000-Biotin) (all from Avanti Polar Lipids, Inc.; Alabaster, AL, USA) were blended in chloroform at a molar ratio of 90:5:5. The chloroform was subsequently removed under nitrogen flow at room temperature. The dried phospholipid blends were hydrated at 60°C with 5 mL phosphate-buffered saline (PBS). Perfluoropropane gas was then added (C3F8; Flura, Newport, TN, USA). These admixtures were mechanically agitated for 45 s to obtain plain microbubbles. Finally, the resulting plain MBs were used to prepare targeted MBs via centrifugal washes, incubation with avidin (Sigma; St. Louis, MO, USA) and conjugation to RGDfK peptides (synthesized by GL Biochem, Ltd., Shanghai, China). Microbubbles bearing RADfK peptides (GL Biochem, Ltd., Shanghai, China) but not RGDfK peptides were prepared as a negative control.

#### Cell Culture

Murine brain microvascular endothelial cells (bEnd.3, which express αvβ3 integrin on their surfaces), and MCF-7 cells (which do not express αvβ3 integrin as a negative control cells) [15] were used to investigate the binding specificity of the RGD-MBs to αvβ3. Both cell lines were purchased from American Type Culture Collection (ATCC, Manassas, VA, USA). Cells were grown in high-glucose Dulbecco’s modified Eagle’s medium supplemented with 10% heat-inactivated fetal bovine serum and 1% penicillin-streptomycin. The cells were maintained at 37°C in a humidified 5% carbon dioxide and 95% air atmosphere.

#### Assessment of Binding Specificity

The bEnd.3 cells and MCF-7 cells were cultured in 6-well plates (1×10^5^ cells/well) overnight to allow cell adhesion. RGD-targeted MBs diluted to different concentrations (1×10^6^, 1×10^7^, 1×10^8^, 1×10^9^ particles/mL, respectively) or control MBs (1×10^8^ particles/mL) were added to the culture wells (following the removal of media) and incubated with the bEnd.3 cells or αvβ3-negative MCF-7 cells for 5 minutes under gentle rotation. Free MBs that did not adhere to the bEnd.3 cells were removed by washing with PBS. The number of attached MBs per field and the number of attached MBs per cell were obtained using an optical microscope (Olympus, Tokyo, Japan) over six random fields of view (200× magnification). For competitive experiments, to block αv integrins, bEnd.3 cells were pre-incubated with 1 ug/ml, 5 ug/ml, or 25 ug/ml anti-alpha(v) antibody for 30 minutes, respectively.

### *In Vivo* Studies

#### Mouse Tumor Model

All animal experiments were approved by the Institutional Animal Care and Use Committee of the People’s Hospital of Guangxi Zhuang Autonomous Region (No. 2012–023). A classic subcutaneous inoculation protocol was used to establish the human laryngeal carcinoma xenograft in nude mice [16]. Briefly, approximately 5×10^6^ Hep-2 cells (Type Culture Collection of the Chinese Academy of Sciences, Shanghai, China) suspended in 0.2 ml PBS were injected subcutaneously into the right flanks of fifteen 6-week-old BALB/c nude female mice (Laboratory Animal Center of Guangdong Province, China). Tumor growth was monitored by ultrasound at least twice weekly, and the maximum diameter of xenografts was allowed to reach 12mm. The mice bearing tumor xenografts were randomly divided into the following three groups based on tumor growth stage: small tumor (tumor volume: 50–150 mm^3^), medium tumor (tumor volume: 151–250 mm^3^), and large tumor (tumor volume: >250 mm^3^).

#### Targeted Contrast-enhanced Ultrasound Imaging

Ultrasound imaging was performed using a dedicated small-animal, high-resolution imaging system (Vevo 2100; VisualSonics, Inc., Toronto, Canada) outfitted with a 40-MHz high-frequency linear transducer. The transmitting acoustic power was set at 30%, the depth was 9–10 mm, and the dynamic range was 30 dB. During the experiment, the mice were anesthetized via intraperitoneal injection of pentobarbital sodium (50 mg/kg, Sigma-Aldrich, St. Louis, MO) and were placed on a heating pad to maintain body temperature. Each tumor was first visualized using conventional B-mode imaging to measure the maximal diameter of the mass. Tumor volume was calculated using the volume formula for an ellipsoid: volume = π/6 × length × width × thickness. To decrease measurement variability, the positions of both the transducer and the animals were initially optimized and fixed throughout the study. After intravenous injection of 5×10^7^ RGD-MBs (100 μL) via the tail vein, the mice were subjected to a 4 min incubation prior to imaging to allow the targeted microbubbles to bind to endothelial molecular markers. Approximately 200 ultrasonographic frames of the tumor tissue and the adherent and freely circulating MBs were then acquired over 10 s. Subsequently, a high-power destructive pulse (0.59 mechanical index, 10 MHz center frequency) was applied for one second to destroy all microbubbles within the ultrasound field. After the destructive pulse, another sequence of 200 images was captured to evaluate the degree of circulating microbubble replenishment.

#### Imaging Analysis

All ultrasound image sequences were digitally recorded and analyzed offline with the software included in the ultrasound scanner. The mean video intensity was measured in regions of interest that encompassed the whole tumor in the imaging plane. To determine the acoustic signal produced by the adherent microbubbles, the difference in video intensity between the pre- and post-destruction imaging frames was calculated and automatically expressed as green code overlays on the B-mode ultrasound images [17].

#### Tumor Immunofluorescence Staining

After being subjected to targeted, contrast-enhanced ultrasound imaging, the mice were sacrificed with cervical dislocation method, and their tumors were harvested. Dissected tumor samples were covered with Tissue-Tek (Sakura, Torrance, CA, USA) and frozen in liquid nitrogen vapor. The samples were serially cross-sectioned into 10-mm-thick slices with a cryostat microtome (CM1950; Leica, Heidelberg, Germany). To visualize ανβ3 integrin expression on tumor endothelial cells, a double immunostaining procedure was performed using a rabbit anti-mouse alpha(v) primary antibody (BD Biosciences, San Jose, CA) at a 1:50 dilution and a FITC-labeled goat anti-rabbit secondary antibody (BD Biosciences, San Jose, CA) at a 1:100 dilution. Cell nuclei were counterstained with 4’,6-diamidino-2-phenylindole (DAPI, Sigma Aldrich, St. Louis, MO). Representative fluorescent images of randomly chosen fields of view were captured using a confocal microscope (Olympus FV1000 confocal microscope; Olympus, Tokyo, Japan).

### Statistical Analysis

SPSS version 13.0 (SPSS, Chicago, IL, USA) was used for data analysis. The data were expressed as the means ± standard deviations. Statistical analysis of microbubbles attachment to cells with and without the addition of blocking antibodies was performed using a two-tailed t-test. The mean video intensities among different tumor groups were analyzed using one-way analysis of variance (ANOVA) and the Student-Newman-Keuls test. Significance was defined as *P* < 0.05.

## Results

### Physical Characteristics of RGD-targeted MBs

**Fig 1:**
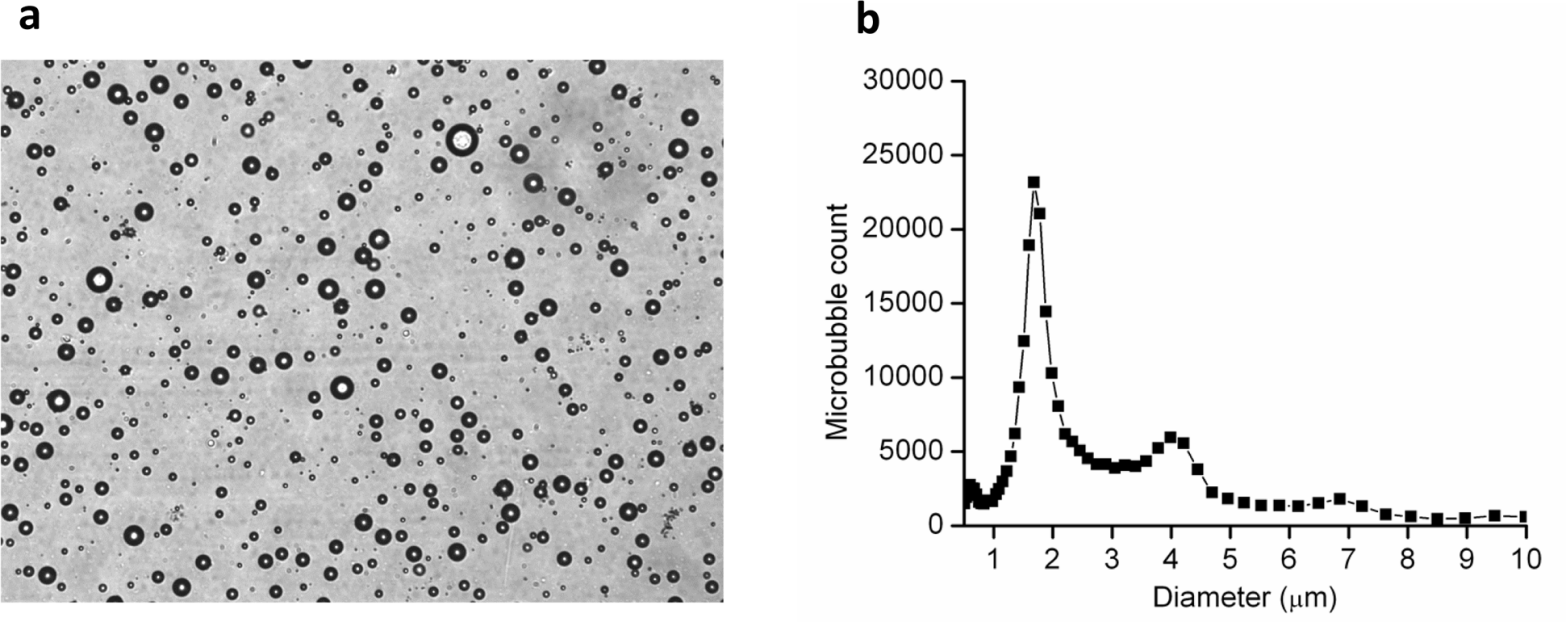
Morphologic characterization and size distribution of RGD-MBs. (a) Representative microscopic image of RGD-MBs (bar = 10 μm). (b) Typical size distribution of RGD-MBs.

The RGD-targeted microbubbles were well dispersed and possessed a spherical appearance under microscopy (Fig 1A). The mean size of the RGD-MBs was 2.36 ± 1.7 μm, and over 95% of the microbubbles were less than 10 μm in diameter. The diameter size distribution was centered at approximately 1.68 μm, with secondary peaks at approximately 0.6 μm, 3.99 μm and 6.85 μm (Fig 1B).

### Binding specificity for RGD-MBs

**Fig 2:**
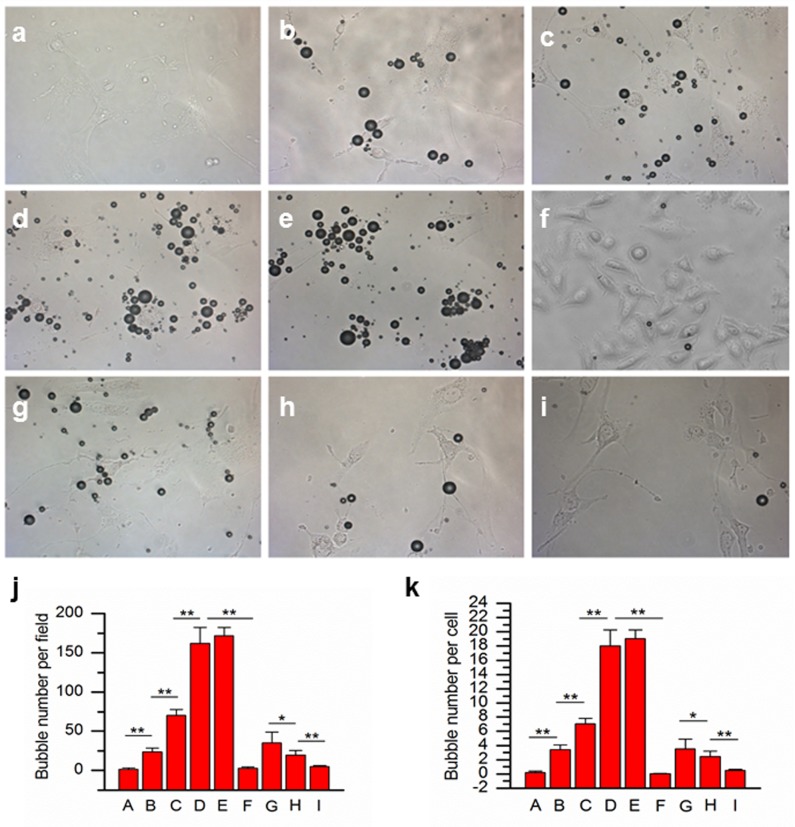
Binding specificity of RGD-MBs. Static binding assay on bEnd.3 cells or αvβ3-negative MCF-7 cells was performed with and without pre incubation with anti-alpha(v) antibodies (×200). (a) Non-targeted control MBs (1×10^8^ particles/mL). (b) 1×10^6^, (c) 1×10^7^, (d) 1×10^8^, and (e) 1×10^9^ particles/mL RGD-MBs. (f) RGD-MBs (1×10^8^ particles/mL) incubated with αvβ3-negative MCF-7 cells. (g-i) RGD-MBs (1×10^8^ particles/mL) with (g)1 ug/ml, (h) 5 ug/ml, and (i) 25 ug/ml anti-alpha(v) antibodies. (j) Quantitative analysis of the number of attached bubbles per field; (k) Quantitative analysis of the number of attached bubbles per cell. (* *P* < 0.05, ** *P* < 0.01).

Table 1 shows the adhesion of different types of microbubbles to bEnd.3 cells or αvβ3-negative MCF-7 cells with and without pre incubation with anti-alpha(v) antibodies. The binding specificity experiment illustrates that few control microbubbles bound to the bEnd.3 cells (Fig 2A). Attachment of RGD-MBs to bEnd.3 cells was significantly higher than the non-modified MB controls (*P* < 0.01). There was rarely binding of RGD-MBs to vβ3-negative MCF-7 cells (Fig 2F). The binding number of RGD-MBs to the bEnd.3 cells was gradually improved with increase of RGD-MBs concentration from 1×10^6^ particles/mL to 1×10^9^ particles/mL (Fig 2B–2E). As expected, when bEnd.3 cells were blocked with anti-alpha(v) antibodies, the number of adherent RGD-targeted MBs was significantly reduced (Fig 2G–2I). This confirms the specific attachment of RGD-MBs to αvβ3 integrin-expressing cells (Fig 2).

**Table 1:** Adhesion of different types of microbubbles to bEnd.3 cells or αvβ3-negative MCF-7 cells with and without pre incubation with anti-alpha(v) antibodies.

Cell line and type of MBs	Adherent MBs per Field		Adherent MBs per Cell	
(particles/mL)	Mean	Standard Deviation	Mean	Standard Deviation
Control MBs (1×10^8^) to bEnd.3 cells	1.667	1.633	0.208	0.204
RGD-MBs to bEnd.3 cells				
1×10^6^	23.833	4.579	3.404	0.654
1×10^7^	70.333	7.763	7.239	1.797
1×10^8^	162	20.317	18	2.257
1×10^9^	171.5	10.784	19.056	1.198
RGD- MBs (1×10^8^) + anti- alpha(V) antibodies to bEnd.3 cells				
1 ug/ml	35.167	13.541	3.517	1.354
5 ug/ml	19.5	6.058	2.438	0.757
25 ug/ml	5	1.414	0.503	0.142
RGD-MBs (1×10^8^) to MCF-7 cells	3	1.789	0.064	0.038

### Targeted, Contrast-enhanced Ultrasound Imaging *In Vivo*

**Fig 3:**
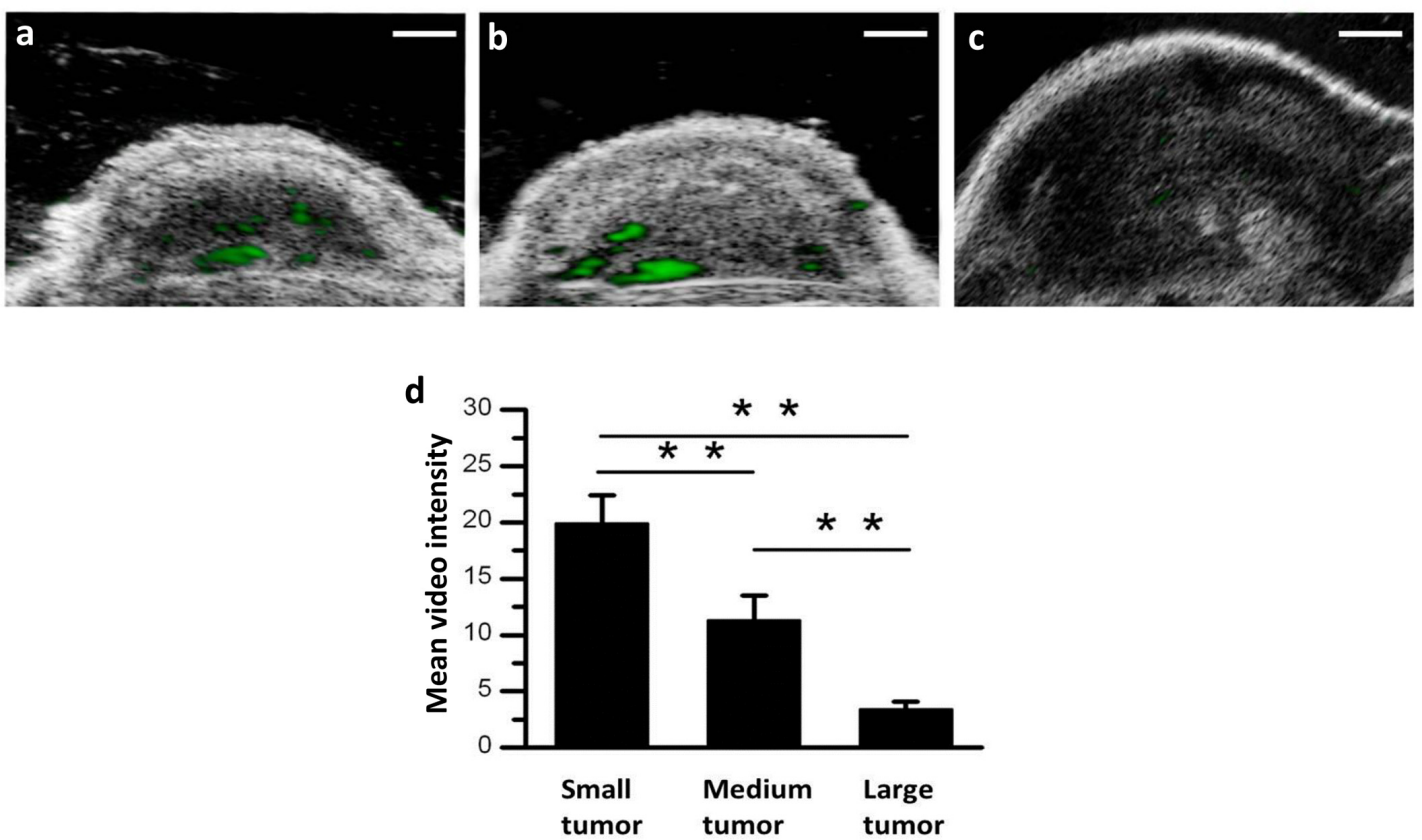
Transverse color-coded ultrasonography after intravenous administration of RGD-MBs obtained at three different tumor stages of subcutaneous Hep-2 tumor xenografts. (a) Small tumor (50–150 mm^3^). (b) Medium tumor (151–250 mm^3^). (c) Large tumor (> 250 mm^3^). Green represents targeted ultrasound signals from adherent RGD-MBs. (d) Quantitative analysis revealed ultrasound imaging signal intensity was highest in the small tumor group (** *P* < 0.01).

There were no signs of acute toxicity following RGD-MB administration in any of the animals. Representative targeted, contrast-enhanced ultrasound images of Hep-2 tumor xenografts at different tumor stages (small tumor, medium tumor, and large tumor) are shown in Fig 3. The quantitative acoustic video intensity following the administration of the RGD-targeted MBs was significantly higher in small tumors (19.89 ± 2.49) than in medium (11.25 ± 2.23) and large (3.38 ± 0.67) tumors. The intensities of the targeted contrast-enhanced ultrasound imaging signals decreased as tumor size increased (Fig 3, *P* < 0.01).

### Tumor Immunofluorescence Assay

**Fig 4:**
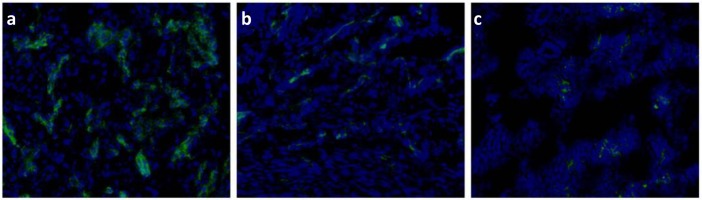
Immunofluorescence staining for αν integrin expression on Hep-2 tumor slices. (a) Small tumor. (b) Medium tumor. (c) Large tumor. Green represents tumor blood vessels; blue represents nuclei stained with DAPI staining (bar = 100 μm).

To validate the above ultrasonography results, the tumors were excised, sliced and stained to visualize ανβ3 integrin expression on tumor neovascular endothelial cells. The expression of ανβ3 integrin in small tumors was significantly higher than in medium and large tumors (Fig 4). This finding was consistent with the results of the targeted signal intensity enhancement obtained at different tumor stages.

## Discussion

Angiogenesis is a critical determinant of solid tumor growth, invasion, and metastasis [18]. At the onset of angiogenesis, a number of pro-angiogenic growth factors and proteolytic enzymes are secreted into the interstitium and cause perivascular basal membrane degradation and smooth muscle cell and endothelial cell proliferation and migration. These events eventually lead to a realignment of the endothelial cells to form new vessels and vascular networks within the tumor [19,20]. Current methods of directly assessing angiogenesis are limited by the following factors: 1) they are invasive and require tissue biopsy, 2) they are susceptible to sampling errors, and 3) they are not feasible for serial monitoring in the clinic [21]. Therefore, the validation and standardization of new noninvasive imaging techniques for assessing angiogenesis and angiogenic phenotypes may provide an important means of analyzing tumor metastatic potential and tumor responses to anti-angiogenic therapies [22].

Contrast-enhanced ultrasound molecular imaging is a particularly attractive method for noninvasive assessment of tumor angiogenesis at the molecular level. In 2003, Ellegala et al. first reported that tumor angiogenesis could be assessed with molecular ultrasound imaging using ανβ3 integrin-targeted microbubbles [22]. Several studies have subsequently reported improved targeting schemes to enhance ultrasound signals to suitable levels for clinical translation.

RGD-targeted microbubbles use small peptide sequences to target ανβ3 integrins with high affinity and specificity. The small sizes of these peptides enable a higher density of microbubbles to target a vascular compartment within a region of interest, thereby enhancing the ultrasound imaging signal [20]. Willmann et al. [23] showed that RGD-targeted microbubbles produced 2-fold stronger imaging signals than those produced by anti-integrin αvβ3 antibody-targeting microbubbles in human ovarian adenocarcinoma xenografts. In a study by Anderson et al. [6], cyclic RGD-targeted microbubbles bound αvβ3 integrin with high specificity and facilitated the quantitative visualization of tumor angiogenesis by contrast ultrasound imaging. However, molecular markers on the surfaces of angiogenic endothelial cells have dynamic expression levels. The usefulness of RGD-targeted microbubbles during ultrasound molecular imaging of early and late stage tumors requires further investigation. In the present study, we reported our initial findings using RGD-targeted MBs prepared via biotin–avidin conjugation to quantify dynamic changes in ανβ3 integrin expression at different stages of tumor growth in human laryngeal carcinoma xenografts.

We further confirmed the specific attachment of RGD-MBs to ανβ3 integrin-expressing cells. We utilized a binding specificity experiment to demonstrate that RGD-MBs exhibited significantly greater adhesion to bEnd.3 cells than did control MBs. The adhesion of RGD-MBs to bEnd.3 cells was significantly inhibited when the cells were pre-treated with anti-alpha(v) antibodies. The mean acoustic video intensity calculated from in vivo ultrasound molecular imaging was significantly higher in small tumors than in medium and large tumors and was well correlated with the results from immunofluorescence analysis. These data suggest that RGD-MBs enable noninvasive longitudinal assessment of changes in tumor angiogenic molecular marker expression levels during tumor growth. Similarly, Deshpande et al. [24] demonstrated that the intensities of targeted, contrast-enhanced ultrasound imaging signals corresponded to the expression levels of endoglin, ανβ3 integrin, and vascular endothelial growth factor receptor (VEGFR)-2. Furthermore, they showed that the expression levels of these molecular markers in human breast, ovarian and pancreatic xenografts were highest in small tumors and decreased as tumors grew. This phenomenon may be relative to the centric necrosis within the large tumors. Another hypothesis was that numerous pro-angiogenic growth factors and proteolytic enzymes secreted into the interstitium highly upregulated both sprouting and intussusceptive angiogenesis in early fast growing tumors, while in larger stabilized tumors frequently observed intussusceptive angiogenesis only [20, 25]. Our results demonstrate that early-stage cancers can be detected using RGD-MBs when tumors are too small to evaluate morphologic changes but are large enough to visualize tumor angiogenesis. The characteristics of angiogenesis at different tumor stages can also be exploited to monitor the effects of anti-angiogenic treatments.

In the current study, a newer generation Vevo 2100 high-frequency ultrasound system specifically designed for small-animal imaging was used. In addition to high spatial resolution, the Vevo 2100 ultrasound system is capable of operating in a contrast-specific imaging mode using uncompressed image signals and can systematically detect nonlinear fundamental and subharmonic signal components. This approach improves the contrast-to-tissue ratio by using a pulse-sequencing method based on amplitude modulation [24, 26].

There are two limitations in the current study. First, a two-dimensional imaging acquisition method was used. Streeter et al. [27] indicated that the use of contrast-enhanced, three-dimensional ultrasound molecular imaging can reduce errors associated with two-dimensional single plane imaging. Furthermore, three-dimensional imaging improves the quality of the collected data and is a thorough means of characterizing angiogenesis. Additional studies based on three-dimensional volumetric imaging are necessary to further validate the use of targeted, contrast-enhanced ultrasound for the longitudinal quantification of molecular marker expression levels. Second, the high-frequency imaging system described in our study allowed the detection of tumor angiogenesis in a field of view that was limited by the transducer beam elevation. Additional investigations may be required to address the differences between low-frequency and high-frequency ultrasonography in the quantitative molecular imaging of tumor angiogenesis.

In conclusion, the results of our study suggest that the use of RGD-based ανβ3 integrin-targeted microbubbles enables noninvasive in vivo visualization of tumor angiogenesis, which was shown to vary during tumor growth in a Hep-2 cancer xenograft.
